# Cognitive biases in first psychotic episode with Attention deficit and hyperactivity disorder: a controlled study

**DOI:** 10.1192/j.eurpsy.2022.535

**Published:** 2022-09-01

**Authors:** N. Manzanares Tesón, M. Solé, M.J. Algora, A. Cabezas, V. Sánchez-Gistau

**Affiliations:** 1 Psychiatrist, Hospital Psiquiàtric Universitari Institut Pere Mata de Reus, Early Intervention Service, reus, Spain; 2 Hospital Psiquiàtric Universitari Institut Pere Mata de Reus, Early Intervention Service, reus, Spain

**Keywords:** adhd, First Psychotic Disorder, cognitive biases

## Abstract

**Introduction:**

Cognitive biases are a core feature of psychotic disorders. Moreover, people with first episode of psychosis (FEP) have more difficulties in social cognition, in particular in theory of mind. On the other hand, deficits in processing speed and distractibility appear to be core features of attention deficit hyperactivity disorder (ADHD) and impairment in these basic processes can lead to deficits in more complex functions, that could induced to cognitive biases.

**Objectives:**

To evaluate whether FEP with and without ADHD differ in the rate and type of cognitive biases.

**Methods:**

Participants 121 FEP treated at the Early Intervention Service of Reus and aged between 14 and 28 years. *Instruments : The Diagnostic Interview for ADHD (DIVA) and the Cognitive Biases Questionnaire for Psychosis (CBQp) measuring 2 themes : anomalous perception (AP) and threatening events (TE) and 5 cognitive biases: Intentionalising (Int) , Catastrophising (Cat), Dichotomous thinking (DT), Jumping to conclusions (JTC) and Emotional reasoning (ER)*

**Results:**

31 out 121 (25.6%) met criteria for childhood ADHD. Compared with FEP ADHD- , FEP-ADHD+ presented significant higher scores in the CBQp total score (U= 2.538 ; p=0.001), the AP theme (U=2.262; p=0.02) , the TE theme (U= 2.242 ; p=0.02) and DT bias ((U= 2.188 ; p=0.03)

**Conclusions:**

Our findings support the fact that subjects with FEP-ADHD+ presented more cognitive biases than those ADHD-. So, FEP-ADHD+ subjects could represent a clinical subgroup with a worse prognosis than FEP-ADHD ^-^ subjects, presenting more delusions, distress and a worse cognitive insight.

**Disclosure:**

No significant relationships.

